# Kruskal-Wallis-Based Computationally Efficient Feature Selection for Face Recognition

**DOI:** 10.1155/2014/672630

**Published:** 2014-05-21

**Authors:** Sajid Ali Khan, Ayyaz Hussain, Abdul Basit, Sheeraz Akram

**Affiliations:** ^1^Department of Software Engineering, Foundation University, Rawalpindi 46000, Pakistan; ^2^Department of Computer Science, Shaheed Zulfikar Ali Bhutto Institute of Science and Technology Islamabad, Islamabad 44000, Pakistan; ^3^Department of Computer Science and Software Engineering, International Islamic University, Islamabad 44000, Pakistan

## Abstract

Face recognition in today's technological world, and face recognition applications attain much more importance. Most of the existing work used frontal face images to classify face image. However these techniques fail when applied on real world face images. The proposed technique effectively extracts the prominent facial features. Most of the features are redundant and do not contribute to representing face. In order to eliminate those redundant features, computationally efficient algorithm is used to select the more discriminative face features. Extracted features are then passed to classification step. In the classification step, different classifiers are ensemble to enhance the recognition accuracy rate as single classifier is unable to achieve the high accuracy. Experiments are performed on standard face database images and results are compared with existing techniques.

## 1. Introduction

Face recognition is becoming more acceptable in the domain of computer vision and pattern recognition. The authentication systems based on the traditional ID card and password are nowadays replaced by the techniques which are more preferable in order to handle the security issues. The authentication systems based on biometrics are one of the substitutes which are independent of the user's memory and not subjected to loss. Among those systems, face recognition gains special attention because of the security it provides and because it is independent of the high accuracy equipment unlike iris and recognition based on the fingerprints.

Feature selection in pattern recognition is specifying the subset of significant features to decrease the data dimensions and at the same time it provides the set of selective features. Image is represented by set of features in methods used for feature extraction and each feature plays a vital role in the process of recognition. The feature selection algorithm drops all the unrelated features with the highly acceptable precision rate as compared to some other pattern classification problem in which higher precision rate cannot be obtained by greater number of feature sets [1].

The feature selected by the classifiers plays a vital role in producing the best features that are vigorous to the inconsistent environment, for example, change in expressions and other barriers. Local (texture-based) and global (holistic) approaches are the two approaches used for face recognition [2]. Local approaches characterized the face in the form of geometric measurements which matches the unfamiliar face with the closest face from database. Geometric measurements contain angles and the distance of different facial points, for example, mouth position, nose length, and eyes. Global features are extracted by the use of algebraic methods like PCA (principle component analysis) and ICA (independent component analysis) [3]. PCA shows a quick response to light and variation as it serves inner and outer classes fairly. In face recognition, LDA (linear discriminate analysis) usually performs better than PCA but separable creation is not precise in classification. Good recognition rates can be produced by transformation techniques like DCT (discrete cosine transform) and DWT (discrete wavelet transform) [4]. To analyze unstable signals, wavelet analysis is used which is fast and also provides good frequency domain quality. Image of the face is divided first into subregions [5]. Afterwards facial features are extracted using weber local descriptor. The orientation component is done by Sobel descriptor. The subregions of an image are recognized by the use of the nearest neighborhood method. Integration in decision level results in final recognition. Rates of recognition are high but costly. In [6] two famous techniques are discussed in order to extract face features. Significant features are selected by particle swarm optimization. It also decreases the dimensions of data and Euclidean distance classifier is trained and tested by using optimized features. But the problem with PSO is that it is an expensive process.

A lot of methods, for example, greedy algorithm [7], branch and bound algorithm [8], mutual information [9], and tabu search [10], have been used on the testing and training data for feature selection. Methods based on genetic algorithm [11] and ant colony optimization have gained a lot of attention [12] which are population-based optimization algorithms. These methods try to provide a good solution by obtaining knowledge from the older iteration. Feature selection algorithm usually uses heuristic in order to avoid confusion.

The main aim of the paper is to introduce a face recognition system that is computationally less expensive, and only the information related to facial features is used. For this DWT- and WLD-based techniques are used to extract face features. The significant features with high information are utilized by Kruskal-Wallis algorithm and the results are compared with the famous techniques like PSO and GA. An ensemble of three classifiers is used to improve the precision rate of recognition.

## 2. The proposed Methodology

**Figure 1:**
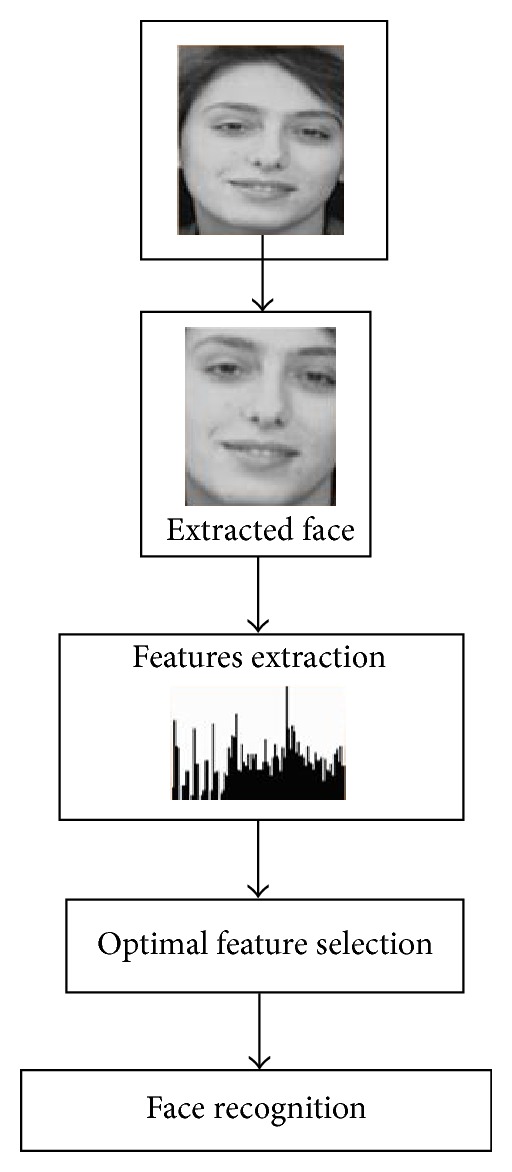
The proposed system architecture.

The proposed techniques steps are represented by Figure 1. In the first step, facial features are extracted using discrete wavelet transform. To reduce the data dimensions, computationally efficient technique (Kruskal-Wallis) is applied to select the most prominent face features. In the last step, different well-known classifiers are trained and tested using those extracted features to recognize the face image.

### 2.1. Feature Extraction

Two techniques are used to extract the face features. Details of these techniques are provided below.

#### 2.1.1. Wavelet-Based Face Feature Extraction

DWT is one of the wavelet transforms which was founded in 1976 when discrete time signals were decomposed by Polikar [13]. It is substitute for cosine transform in which the functions of sine cosine are added and the varying value of time and frequency is returned by wavelet.

**Figure 2:**
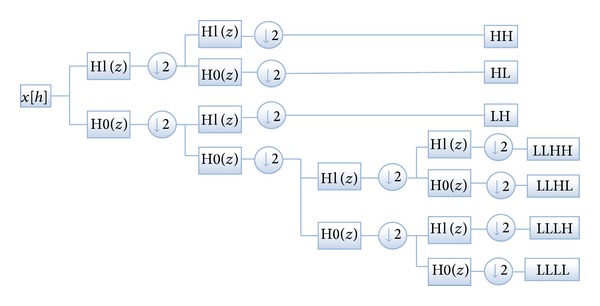
Standard DWT [13].

Decomposition by columns and rows is the second famous method which uses low pass filter in its iterations. The input image is divided into subcomponents after passing through low and high pass filter. These subbands include the details about vertical and horizontal properties of an image. The low pass filter gives low frequency subbands which includes detailed information and the process is repeated on the subband in the second level. The high pass filter gives the high frequency subbands, the vertical subbands, and the diagonal coefficients.  Figure 2 shows DWT standard method.

**Figure 3:**
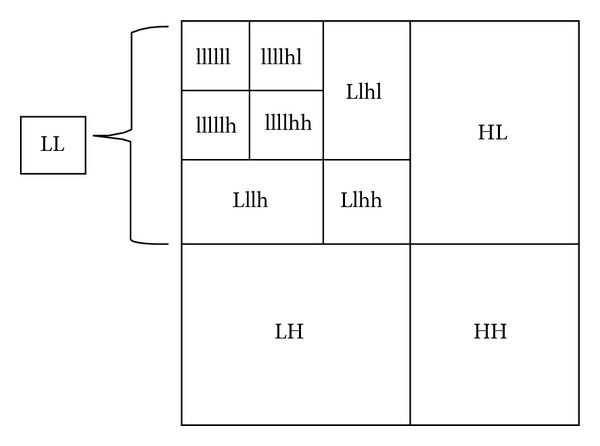
Standard 2D DWT decomposition.

Figure 3 shows the detailed process of DWT. Four subbands of an image (LL, HL, LH, and HH) are acquired by applying DWT. LL shows the approximate coefficients. Detail coefficients are represented by HL, LH, and HH. There are different types of wavelet transform, for example, Symmlet, Haar, Daubechies, and Coiflet with various numbers of vanishing moments (features of wavelets). These are scaling functions which show complex signals precisely [14].

Daubechies wavelet is used in the proposed method to extract DWT features. It is the orthogonal wavelets which show the discrete wavelet transform with greater vanishing moments. Another scaling function is named as father wavelet which produces an orthogonal multiresolution analysis (MRA) used in the method [15]. The scaling function makes sure that the whole spectrum is covered and filters the lowest level of transform. The MRA is the sequence of nested subspaces. Vector space is the first element of the MRA and for every vector space there exists another vector space with higher resolution until a final image is obtained.

### 2.2. Feature Selection

The performance of classification system can be degraded by using all the features of input data as it increases the complexity. Picking up the optimized features is very important as some features play more important role in recognition. There are a lot of methods that are developed and have been used for features but most of them are computationally expensive and complex in nature. Kruskal-Wallis method [16] is used in the proposed method in order to select significant features which is computationally less expensive and very simple in use. Kruskal-Wallis method tests if two or more classes have equal median and gives the value of *P*. Features with discriminative information are selected. If the value of *P* is close to “0” it means that the feature contains discriminative information; otherwise it will not be selected. DWT features are processed using the Kruskal-Wallis technique. Features which result in a value of *P* less than a threshold are selected to be used in the next recognition step.

### 2.3. Classification

**Figure 4:**
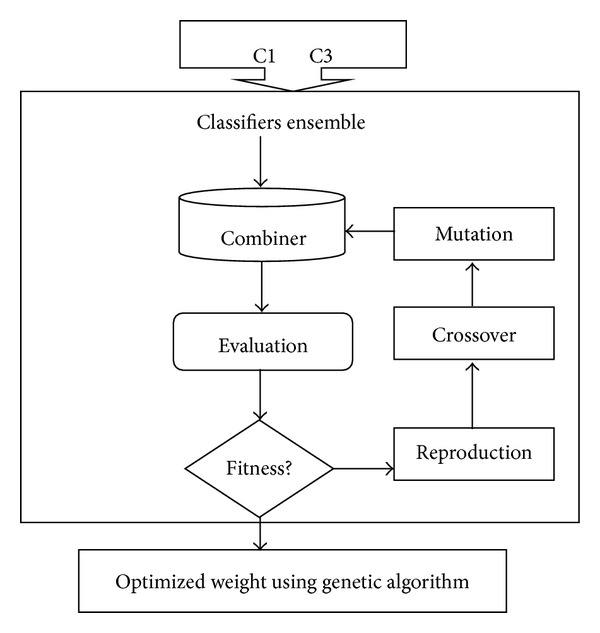
Classifiers ensemble and optimization process flow diagram.

Single classifier is unable to achieve high accuracy rate so two well-known classifiers are trained and tested.  Figure 4 represents the classifiers ensemble and optimization process using Genetic algorithm. Description of the classifiers is given below.

#### 2.3.1. *K*-Nearest Neighbour Classifier

*K*-nearest neighbour classifier classifies the sample data by allocating it to that class label which more commonly represents its nearest neighbours value, that is, *k*. If the tie situation occurs between test samples then decision is based on distance calculation. The sample will be assigned to that class which has smaller distance from the test sample. KNN performance is dependent on the optimal value of *K* and the distance. Different methods have been used by researchers to calculate the distance, for example, Euclidean, Minkowsky, and cambra. The Euclidean distance method is more common and famous [17]. The equation used to calculate the distance between the two points, *X* and *Y*, is as follows:

(1) $$\begin{array}{c} d\left(X,Y\right)=\sqrt{\overset{n}{\underset{i=1}{\sum }}{\left(x_{i}-y_{i}\right)}^{2}}, \end{array}$$

where *X* = {*x*_1_, *x*_2_, *x*_3_,…, *x*_*n*_) and *Y* = {*y*_1_, *y*_2_, *y*_3_,…, *y*_*n*_).

Learning speed of KNN classifier is fast but its classification accuracy is relatively poor. KNN has another beauty that it is the smallest classifier compared to all other machine learning algorithms [17].

#### 2.3.2. Support Vector Machine (SVM)

SVM assigns the testing samples to the class whose distance is maximum to the nearest point in the training set. SVM draws a hyperplane if the data is linearly separable. Distinct kernel is utilized to check the accuracy of SVM [18].

#### 2.3.3. Optimization through GA

The classifier behaviour changes every time, and it is possible that the accuracy of a better classifier may degrade and vice versa. Some mechanism needs to be developed to keep the classifier accuracy rate at an acceptable rate. In our approach, the weights of the classifiers are optimized using genetic algorithm to achieve this goal. GA is used in many distinct optimization concepts because it does not require any particular knowledge about problem domain. First, the GA uses problem space to randomly pick different solutions, that is, *N*. In each iteration, selection and reproduction operator are used to optimize these problems.

We normalize the weights between [0-1]. The chromosome “*m*” length is matched to the number of classifiers used, that is, *l*. The results produced by the classifiers are used as initial population and then error rate is generated after applying the fitness function for evaluation. Elitism policy is used in the experiments to bring the “*e*” number of the best chromosome in the new generation. In the next step, new weights are found using mutation “mu” and crossover operation “cr.”

The condition that whether the new weight should be given to the classifiers or not is the quality of the chromosomes. The GA will terminate when the generation reached to Gmax or population convergence to fulfill solution [19].

## 3. Experimental Results and Discussion 

**Figure 5:**
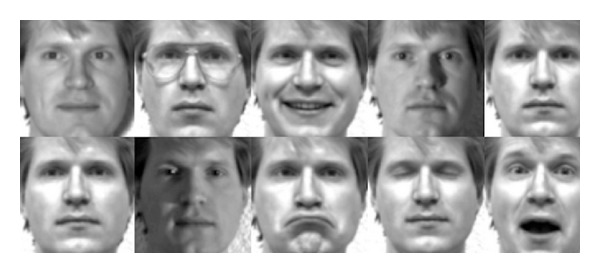
Extended Yale face database sample images.

We have performed experiments on extended Yale face database *B* [20]. Yale database contains 16,128 images of 28 different individuals in GIF format and of size 100 × 100. These variations in expressions include sleepy, sad, wink, and happy. Light conditions are also changed which include normal light, centre light, and right light.  Figure 5 shows the Yale face database sample images. Leave-one strategy is used in all experiments and performance of the system was evaluated and compared after performing different steps.

First, the DWT-based coefficients are extracted from the face. The image size is reduced to 1/4 after implementation of 2D discrete wavelet transform. The image is decomposed up to two levels and different feature vectors are formed.  Figure 2 depicts the 2-level DWT decomposition strategy.

**Figure 6:**
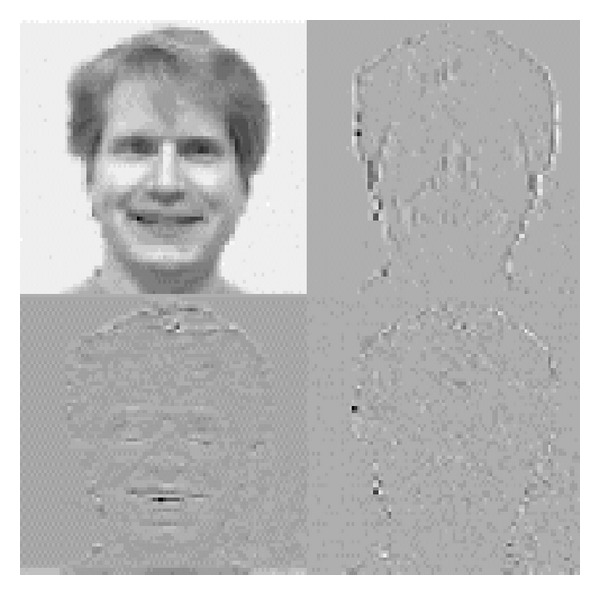
1-level DWT decomposition.

In Figure 6, the top right corner image represents the image having maximum discriminative information and more stable as compared to other subbands. We have used this subband for the next level decomposition. Then after the second level decomposition using Daubechies wavelet, the most important features are extracted by applying principal component analysis (PCA). The features having higher eigenvectors are selected. The feature vector dimensions are 11 × 14, 9 × 8, and 6 × 8, which are correlated to the levels 0, 1, 2, and 3 in decomposition of wavelet.

Table 1 presents the recognition accuracy rate of DWT-based extracted features using Daubechies family.

During the experiments, different classifiers are trained and tested using the extracted features. Performance of KNN and SVM is better than other classifiers in case of classifying face images. In case of KNN, accuracy rate of 88% is achieved using feature vector of size 11 × 14. Accuracy rate increases when the feature vector size decreases (i.e., 9 × 8). The best accuracy of 91% is obtained using KNN in case of 9 × 8 feature vector size.

Table 2 presents the results after using Haar family of DWT. We have observed that in our case the Daubechies family of wavelet performed slightly better than Haar wavelet. It has been observed that SVM is outperformed as compared to KNN using feature set of different sizes. Average accuracy rate of 92% is obtained using SVM classifier.

After performing different experiments, we noted that some of the face features are redundant and do not contribute to recognition process. Kruskal-Wallis feature selection technique is used to select discriminative face features. In Kruskal-Wallis algorithm the value of *P* is changed to eliminate the redundant features and find the optimum threshold. The variation [0.01~0.19] and [2.0 of 4.0] of *P* was followed for the experiments. After implementation of Kruskal-Wallis algorithm feature vector of size 40 is obtained. These features contained more discriminative information about face and produced high recognition rate.

Single classifier is unable to achieve the highest accuracy rate. In the next step, SVM and KNN classifier is ensemble and optimized using GA to improve the recognition rate.

**Figure 7:**
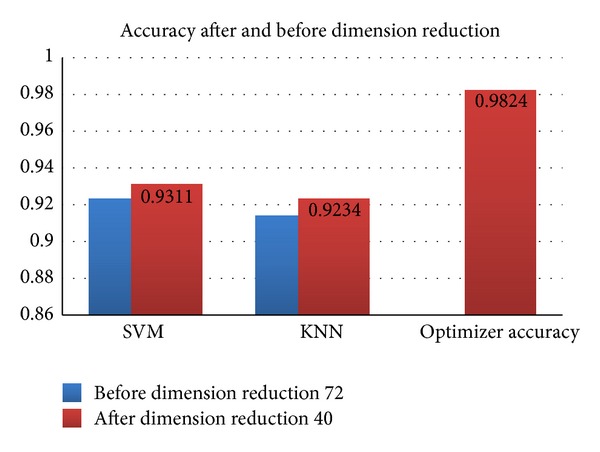
Different classifiers accuracy rate before and after data dimension reduction.

Figure 7 represents the recognition rate of single classifier and accuracy after the classifiers are ensemble and optimized by GA. It has been observed that data of high dimension and irrelevant feature decrease the accuracy rate and also are time consuming. After optimization process the average accuracy rate of 98% is achieved.

In Table 3, the proposed technique is compared with other existing techniques in terms of recognition rate accuracy.

## 4. Conclusions and Future Work

In this work, DWT is analyzed to extract the prominent face features. The search space is reduced greatly by using Kruskal-Wallis algorithm. Kruskal-Wallis algorithm selects the more discriminative face features. It is concluded that single classifier is unable to achieve the high accuracy rate. In order to improve the accuracy rate, two well-known classifiers are ensemble and then optimized using GA. After optimization process the accuracy rate increases. Kruskal-Wallis algorithm searching strategy is simple and less time consuming as compared to other GA and particle swarm optimizations.

In the future the proposed technique will be modified and will be used for 3D face images.

## Figures and Tables

**Table 1:** Face recognition accuracy rate on DB wavelet.

Classifier/features	11 × 14	9 × 8	6 × 8
KNN	0.884	0.914	0.8332
SVM	0.8571	0.899	0.8134

**Table 2:** Face recognition accuracy on Haar Wavelets.

Classifier/features	11 × 14	9 × 8	6 × 8
KNN	0.866	0.914	0.827
SVM	0.871	0.9233	0.844

**Table 3:** Proposed technique comparison with existing techniques.

Method	Recognition rate
Proposed technique	98%
DWT + PSO [6]	96%
Local ternary pattern (LTP) [21]	91%
K2DSPCA [22]	96%
Harmony search algorithm [23]	94%
SIFT [24]	91%
